# Force depression following a stretch-shortening cycle is independent of stretch peak force and work performed during shortening

**DOI:** 10.1038/s41598-018-19657-8

**Published:** 2018-01-24

**Authors:** Rafael Fortuna, Hannah Kirchhuebel, Wolfgang Seiberl, Geoffrey A. Power, Walter Herzog

**Affiliations:** 1 Human Performance Laboratory, Faculty of Kinesiology, University of Calgary, Calgary, Canada; 2 Department of Biomechanics in Sports, Faculty of Sport and Health Sciences, Technische Universitaet Muenchen, Munich, Germany; 3 Neuromechanical Performance Research Lab, Department of Human Health and Nutritional Sciences, College of Biological Sciences, University of Guelph, Guelph Ontario, Canada

## Abstract

The steady-state isometric force following active muscle shortening or lengthening is smaller (force depression; FD) or greater (residual force enhancement; RFE) than a purely isometric contraction at the corresponding length. The mechanisms behind these phenomena remain not fully understood, with few studies investigating the effects of FD and RFE in stretch-shortening cycles (SSC). The purpose of this study was to investigate the influence of RFE and peak force at the end of the stretch phase on the steady-state isometric force following shortening. Isometric thumb adduction force measurements were preceded by an isometric, a shortening contraction to induce FD, and SSCs at different stretch speeds (15°/s, 60°/s, and 120°/s). The different peak force values at the end of stretch and the different amounts of work performed during shortening did not influence the steady-state isometric force at the end of the SSC. We conclude that the FD following SSC depends exclusively on the amount of RFE established in the initial stretch phase in situations where the timing and contractile conditions of the shortening phase are kept constant .

## Introduction

Residual force enhancement is defined as an increase in the steady-state isometric force following active stretching compared to the corresponding force of a purely isometric contraction at the same muscle length^1–3^. Previous studies have shown that RFE depends on the magnitude of stretching^1,2,4^ and is independent of the stretch velocity^5^. RFE has been attributed to changes in cross-bridge kinetics^6^, either through an increase in the proportion of attached cross-bridges or an increase in the average force per attached cross-bridge^7^. RFE has also been thought to be caused by the engagement of a passive structural element upon activation^8–13^. Titin has been implicated as this passive structural element by either increasing its spring stiffness or by reducing its free spring length^11–13^.

Force depression is defined as a decrease in the steady-state isometric force following active shortening compared to the purely isometric force at the corresponding muscle length^1,14,15^. Force depression has been shown to depend on the magnitude of shortening^15,16^ and the amount of work performed during the shortening phase^14,17^. Marechal and Plaghki (1979) proposed that FD is associated with a reduction of the proportion of attached cross-bridges because of a stress-induced deformation of the actin filaments entering the myofilament overlap zone in the shortening phase. This theory is consistent with the reduction in muscle stiffness observed in the FD state compared to purely isometric reference contractions^18^, and the maintenance of ATP consumption per unit of force in the FD state^19^.

Investigations into the mechanisms of RFE and FD are usually performed separately, with few studies combining stretching and shortening (Herzog & Leonard 2000; Rassier & Herzog 2004; Seiberl *et al*.^20^). However, during activities of daily living, muscles undergo stretch-shortening cycles (SSC) on a regular basis. Furthermore, stretch preceding shortening and shortening preceding stretch have revealed muscle properties that cannot be explained based on findings of pure stretch or pure shortening experiments.

Herzog and Leonard showed in cat soleus muscles that stretching followed by shortening had no effect on FD observed after the SSC^16,17^. In contrast, Seiberl *et al*. reported in human adductor pollicis muscle that FD was decreased or even abolished if active shortening was preceded by an active stretch^20^. Furthermore, Fortuna *et al*. showed that stretch induced RFE preceding shortening affects the steady-state isometric force (FD) in a time- and speed-dependent manner^21^. Fast shortening speeds, or shortening immediately following the stretch led to an increase in the steady-state isometric force compared to the force obtained for pure FD tests, thereby causing less FD^22^. However, if shortening following a stretch is delayed by about 1 s or more, or the shortening speed is “slow”, force depression similar to that seen in pure shortening contractions is observed^22^. These observations led to the conclusion that during a SSC shortening-induced force depression does not depend on the work performed during shortening or the force prior to shortening, as has been assumed to date for pure shortening contractions, but may depend exclusively on the amount of force enhancement produced in the initial stretch phase; that is, force depression is offset by the force enhancement achieved in the stretch phase.

Therefore, the purpose of the present study was to investigate the changes in FD in stretch-shortening cycles where the stretch phase produced similar amounts of residual force enhancement, but different peak forces at the end of stretch, thus presumably leading to differing amounts of work in the shortening phase following active lengthening. We hypothesized that FD would be similar for all tests as the amount of force enhancement in the stretch phase was deliberately made to be constant while peak forces following the stretch phase (and thus presumably work) were systematically altered. In order to achieve this goal, the same shortening contractions (same shortening speed and magnitude) were preceded by stretches of equal magnitude (thus giving similar amounts of residual force enhancement) but different stretch speeds (thus giving different peak forces).

## Methods

### Participants

Twelve healthy adults gave free informed consent to participate in this study (8 male and 4 female; age 26 ± 2 years; height 172 ± 8 cm; weight 67 ± 9 kg). All subjects were free of neuromuscular disorders and had no injury on the left hand. All experimental procedures were approved by the Conjoint Ethics Committee of the University of Calgary and were conducted in accordance with the principles of the Declaration of Helsinki.

### Experimental setup

For measuring thumb adduction forces and carpometacarpal angular displacements, a custom-designed dynamometer was used^5,23,24^. Subjects sat on an adjustable chair with the arm slightly abducted and the elbow joint flexed at 90°. The left hand was immobilized with a reusable clinical cast (Ezeform, Rehabilitation Division, Smith & Nephew Inc., Germantown, WI) and was secured with two inelastic straps; one at the middle of the forearm, the second in the area of the metacarpal bones. Movement of the wrist and fingers was restricted, except for ab/adduction of the thumb. A rotary stepper motor (Model TS42BP10 Parker Hannifin Corp., Cleveland, OH, USA) was connected to an aluminum rod (1.5 cm diameter and 15 cm long) via gears (1:4 gears ratio). An auxiliary piece for thumb placement and fixation was attached to the end of the rod. During the experiment, the thumb pressed against the auxiliary piece that was in line with the direction of thumb ab/adduction. Thumb adduction forces were obtained through two pairs of calibrated strain gauges (Model CEA-06–0125UN-350, Measurement Group, Inc. Raleigh, NC, USA). Alignment of the carpometacarpal joint with the center of rotation of the motor was ensured to avoid slipping of the thumb during the testing procedures. The left hand was slightly externally rotated for movements of the thumb towards the third finger. Thumb angle was measured using an analog encoder (Series 03 rotary transducer; Hohner Corp., UK). A digital controller (Model Gemini GT6-L8 Digital stepper driver/controller, Parker Hannifin Corp.) controlled the rod/thumb angle. For each subject, the 0° adduction reference angle was obtained as the smallest adduction that could be reached without the dynamometer touching the cast/hand.

### Electrical stimulation

Two self-adhering Ag-AgCl surface electrodes (2 × 3 cm) were placed over the ulnar nerve to electrically stimulate the adductor pollicis muscle. The cathode was set 2 cm proximal to the metacarpal bones on the medial wrist and the anode 2 cm proximal to the cathode. A Grass S8000 stimulator (Astro Med Inc., Lingueil, Quebec Canada) was used to increase stimulation intensity until no further increase in twitch force amplitude was observed (single 100µs square-wave pulses). The last stimulation setting was used to determine maximum voluntary contraction (MVC) using the interpolated twitch technique^25,26^. All subsequent contractions were electrically evoked. The voltage of the nerve stimulation was raised (fixed frequency: 50 Hz) until the evoked force reached 50–60% of the participant’s MVC. The adjusted voltage intensity was applied for 7 s stimulations for all experimental contractions.

### Protocol

**Figure 1:**
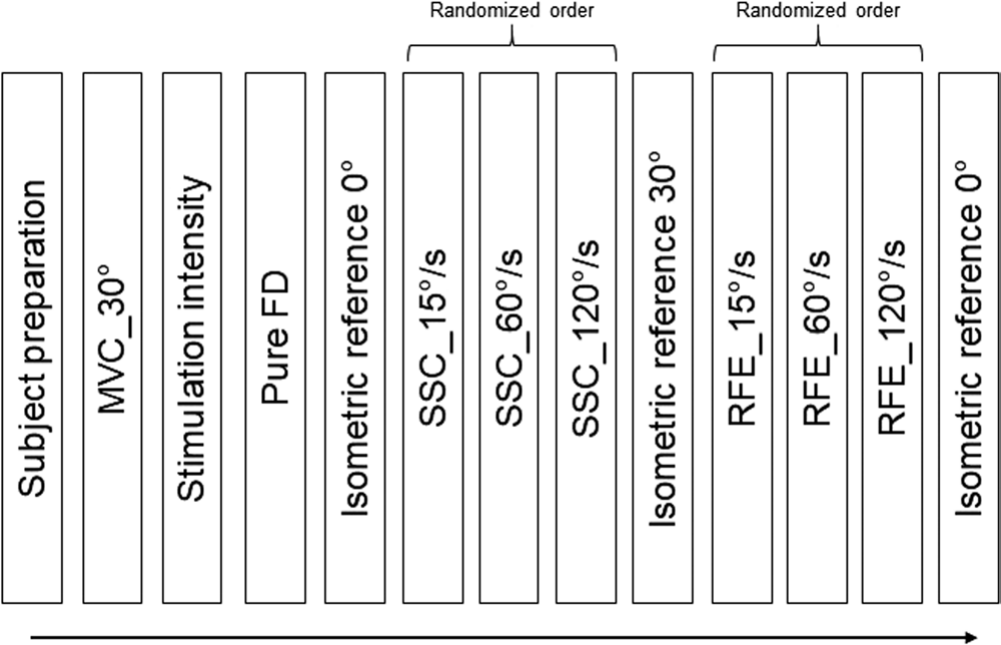
Experimental design of the study. Following participant preparation, a maximum voluntary contraction (MVC) was performed at a 30° thumb abduction angle and the stimulation intensity was then adjusted to produce 50–60% of the MVC force. Next, a force depression (FD) test at 60°/s shortening velocity and a corresponding isometric reference contraction at 0° were performed. Stretch-shortening cycles with varying stretch velocities (15°/s, 60°/s, 120°/s) and a given shortening velocity (60°/s) and shortening magnitude (30°) followed in a randomized order. Then, an isometric reference contraction at 30°, followed by the randomized RFE tests at varying stretch velocities (15°/s, 60°/s, 120°/s) were performed. Last, an isometric reference contraction at 0° was performed to assess muscle fatigue.

Subjects were verbally encouraged to produce a 3–5 s isometric MVC at a 30° thumb abduction angle. Next, the nerve stimulation intensity was adjusted to produce 50–60% of each individual’s MVC. All participants started with a pure shortening test to assess the amount of force depression (FD) unaffected by stretching: the muscle was pre-activated isometrically for 2 s at a thumb abduction angle of 30° followed by a 30° joint excursion at 60°/s shortening velocity. The muscle was then held isometrically at 0° for 4.5 s. Following the FD test, a purely isometric reference contraction at 0° was conducted for 7 s. Next, the stretch shortening cycle (SSC) tests were performed. The muscle was pre-activated and held isometrically at 0° for either 0.5 s, 2.0 s, or 2.25 s and was then stretched to 30° at either 15°/s, 60°/s or 120°/s (SSC_15°/s, SSC_60°/s, and SSC_120°/s, respectively). Following stretching, the muscle was immediately shortened back to 0° at 60°/s for all three SSC trials and then held isometrically for 4.5 s. The order of the SSC conditions was randomized. Subsequently, participants performed an isometric reference contraction at a 30° thumb abduction angle. The next block of tests consisted of pure stretch tests to assess the amount of residual force enhancement (RFE) and peak force for the different stretch conditions. In these tests, the adductor pollicis was held isometrically at a thumb abduction angle of 0° for either 0.5 s, 2.0 s, or 2.25 s and stretched to 30° at either 15°, 60°, or 120°/s (RFE_15°/s, RFE_60°/s, RFE_120°/s, respectively). Finally, we performed another isometric reference contraction at a 0° thumb angle to evaluate possible muscle fatigue. A minimum of 5 min rest was given between each trial (Fig. 1).

### Data reduction and analysis

**Figure 2:**
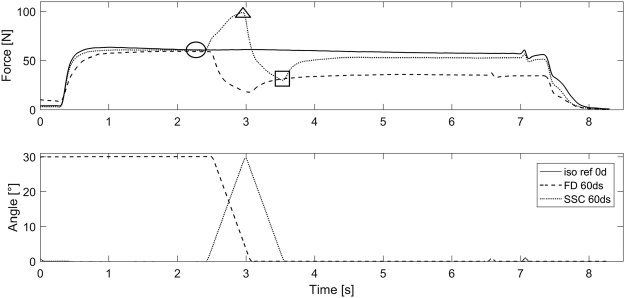
Thumb adduction force (top) and metacarpophalangeal joint angle (bottom) as a function of time for an isometric reference contraction (solid line, Ref_Isometric), a pure shortening-induced force depression (dashed line, FD_60°/s) and a stretch-shortening-cycle at 120°/s (dotted line, SSC_120°/s). The force before stretching (b-STR; •), the maximum force at the end of the stretch (e-STR; ▲), the minimum force at end of shortening (e-SHO; ■) and the average isometric steady-state force (500 ms) prior to muscle deactivation were assessed.

Thumb adduction force and thumb joint angle were sampled at 2000 Hz and collected via an analog-to-digital converter (Windaq, DATAQ Instruments, Inc. USA). Force data were low pass filtered with a cutoff frequency of 10 Hz. Force before stretching (200 ms average: b-STR), peak force at the end of the stretch (maximum force; e-STR), minimum force at the end of shortening (minimum force; e-SHO), and the mean force during a 500 ms period prior to muscle deactivation were used for statistical analysis (Fig. 2). Additionally, the work performed during the shortening phase for pure shortening (FD) and the SSCs was calculated as the line integral of thumb adduction force and thumb displacement. The mean values for the SSCs were compared to the corresponding values obtained in the isometric reference contractions and/or the pure shortening (FD) contractions.

Data were tested for normality (Shapiro-Wilk test) and equal variance (Levene’s test of homogeneity). A one-way repeated measures ANCOVA, using the work performed during shortening as a covariate, with Bonferroni post-hoc correction, was used to test for differences in the steady-state isometric forces following SSCs for the different stretch speeds (SSC_15°/s, SSC_60°/s, and SSC_120°/s) compared to the pure FD (FD_60°/s) and the reference isometric contraction, the peak force at the end of stretching (e-STR) among the different stretch speeds (15, 60, and 120°/s), the minimum force at the end of shortening (e-SHO) for the different stretch speeds (15°/s, 60°/s, and 120°/s), the mechanical work of shortening between the SSCs (SSC_15°/s, SSC_60°/s, SSC_120°/s), and the pure FD (FD_60°/s). Additionally, a linear correlation coefficient was used to assess the relationship between FD and RFE. Statistical significance was set at α = 0.05.

## Results

The mean electrically evoked tetanic isometric force at the 0° thumb angle was 41.0 N ± 3.2 N, representing 58% of the subjects’ MVC (70.6 N ± 4.4 N). There was no significant difference in isometric thumb adduction force at the beginning compared to the end of the testing protocol.

**Figure 3:**
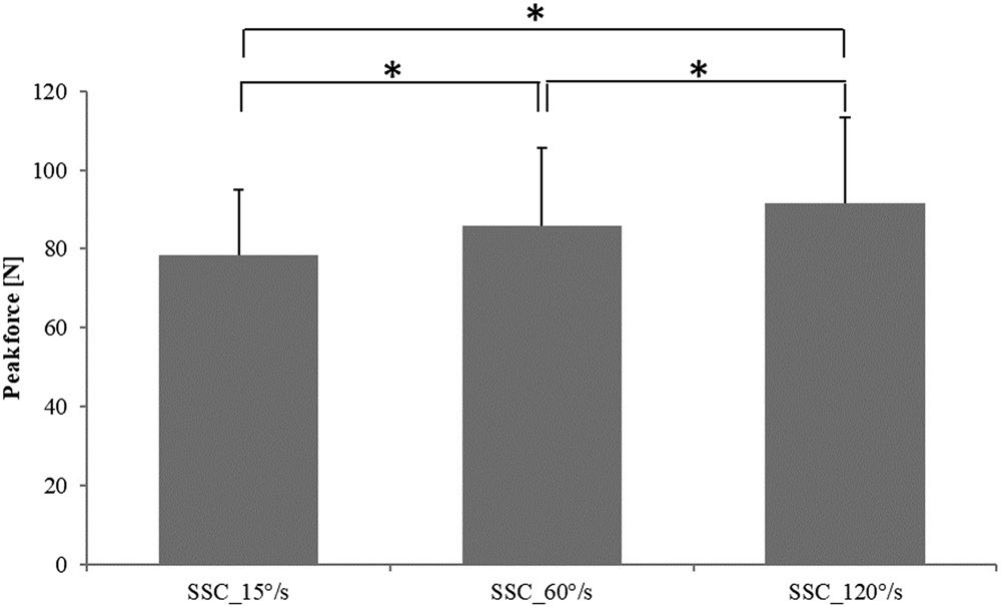
Mean (±SE) values of peak force at the end of the stretch for the SSCs performed at different stretching speeds (SSC_15°/s, SSC_60°/s, and SSC_120°/s). There was a significant increase in peak force for increasing stretching speeds (p < 0.001).

**Figure 4:**
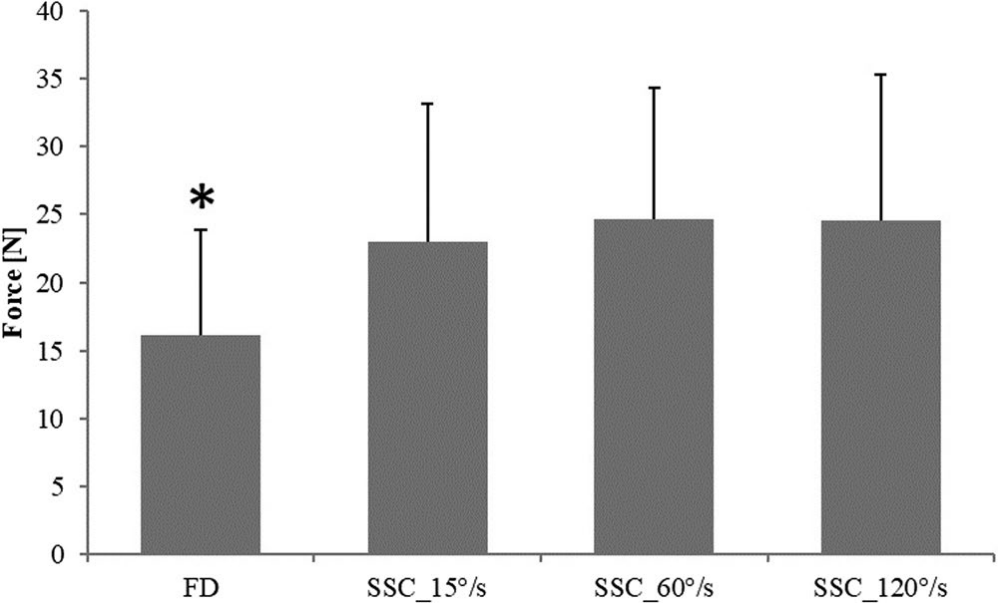
Mean (±SE) values of minimum force (N) for the pure FD test and the SSC tests performed at different stretching speeds (SSC_15°/s, SSC_60°/s, and SSC_120°/s). There was a significant difference in the minimum force for pure FD compared to all SSCs, but there was no significant difference across the SSCs. (*Compared to SSCs; p < 0.001).

**Figure 5:**
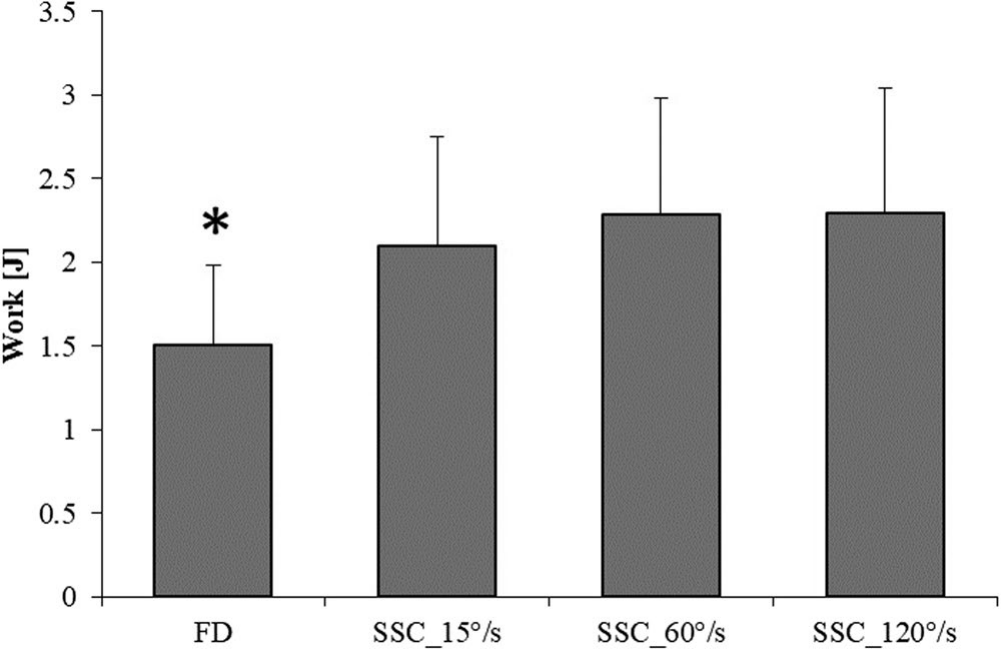
Mean (±SE) values of work performed during shortening for the pure FD test and the SSC tests performed at different stretching speeds (SSC_15°/s, SSC_60°/s, and SSC_120°/s). The work performed during shortening for all SSC was significantly greater compared to that measured for the pure FD test. (*Compared to all SSC; p < 0.001).

There was no difference in force before stretching (b-STR) across the different contractions (not shown). As expected, the fastest stretch velocity (120°/s) produced the greatest peak stretch force (91.7 N ± 6.2 N), followed by the intermediate (60°/s; 85.8 N ± 5.7 N), and the slowest stretch velocity (15°/s; 78.4 N ± 4.8 N) (F(2,22) = 16.00, p < 0.001) (Fig. 3). Force at the end of shortening (e-SHO) was significantly higher (F(3,33) = 19.45, p < 0.001) for all SSC (23.0 N ± 2.9 N, 24.6 N ± 2.7 N, 24.6 N ± 2.7 N for SSC_15°/s, SSC_60°/s, and SSC_120°/s, respectively) compared to the pure FD contraction (16.1 N ± 2.2 N) (Fig. 4). Lastly, work performed during shortening was significantly (F(3,33) = 26.73, p < 0.001) smaller for the pure FD tests (1.5 J ± 0.4 J) compared to the SSC tests (2.0 J ± 0.6 J, 2.2 J ± 0.6 J, 2.2 J ± 0.7 J for SSC_15°/s, SSC_60°/s, and SSC_120°/s, respectively) (Fig. 5). An ANCOVA was performed to investigate the effects of the work performed during shortening (covariate) on the steady-state isometric force following SSCs. There was no statistically significant relationship between the steady state isometric force following SSCs and the work performed during shortening (F(3,29) = 0,45, p = 0.72).

**Figure 6:**
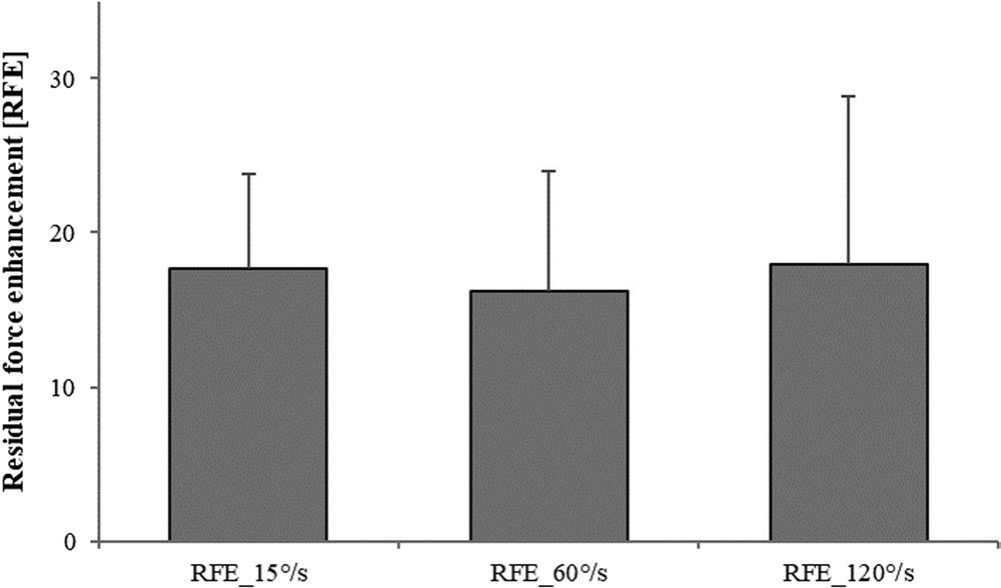
Mean (±SE) values of residual force enhancement for the varying stretching speeds (RFE_15°/s, RFE_60°/s and RFE_120°/s) normalized to the values of the isometric reference contraction at the corresponding thumb angle. Residual force enhancement values were similar (p > 0.05) across the different stretch speeds (17.7% ± 1.7%, 16.2% ± 2.2%, 17.9% ± 3.1% for RFE_15°/s, RFE_60°/s, and RFE_120°/s, respectively).

**Figure 7:**
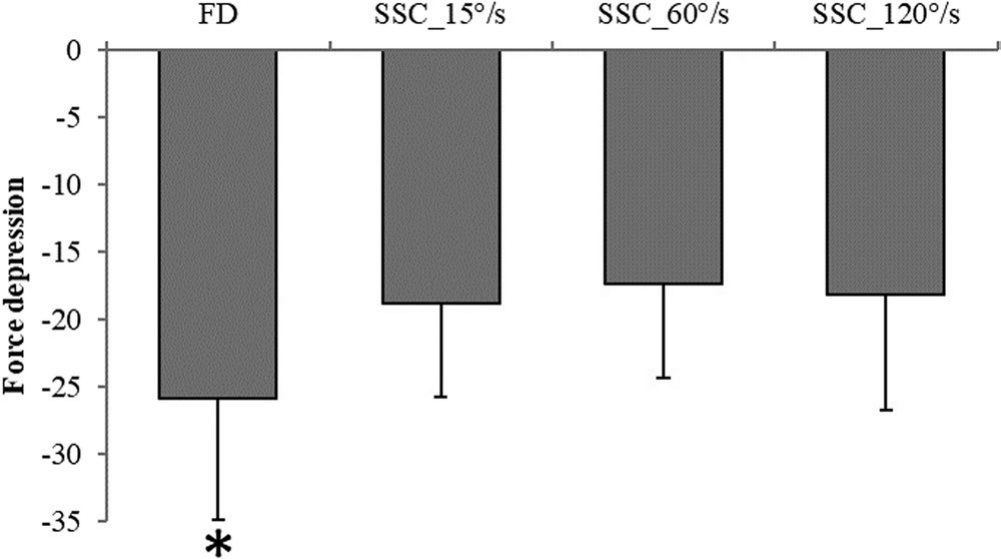
Mean (±SE) values of force depression for pure FD tests (FD_60°/s) and for SSC tests performed at 15°/s, 60°/s and 120°/s (SSC_15°/s, SSC_60°/s, SSC_120°/s, respectively) normalized to the values of the isometric reference contraction at the corresponding thumb angle. Force depression without prior stretching was 25.9% ± 2.5% and was significantly greater compared to all SSCs (18.8% ± 2.0%, 17.4% ± 1.9%, 18.1% ± 2.4% for SSC_15°/s, SSC_60°/s, and SSC_120°/s, respectively). However, no significant difference was found between SSCs. (*Compared to all SSC; p < 0.05).

The steady-state isometric force following active stretching (the residual force enhancement) was the same across all stretch velocities, as expected (17.7% ± 1.7%, 16.2% ± 2.2%, 17.9% ± 3.1% for SSC_15°/s, SSC_60°/s, and SSC_120°/s, respectively) (F(2,20) = 1.86, p = 0.181; Fig. 6). A correlation coefficient was computed to assess the linear relationship between FD and RFE. There was no statistically significant relationship between FD and any of the RFE conditions (RFE_15°/s (r = 0.052, p = 0.871); RFE_60°/s (r = 0.291, p = 0.386), and RFE_120°/s (r = 0.332, p = 0.292). Additionally, the steady-state isometric force was significantly (F(3,31) = 3.98, p = 0.017) depressed by 25.9% ± 2.5% following active shortening (FD) compared to the isometric reference contraction at the corresponding thumb angle (Fig. 7). The steady-state isometric forces following SSCs were significantly greater compared to the pure FD tests (18.9% ± 2.0%, 17.4% ± 1.9%, 18.1% ± 2.4% for SSC_15°/s, SSC_60°/s, and SSC_120°/s, respectively vs 25.9% ± 2.5% for the pure FD) (Fig. 7).

## Discussion

The present study was designed to investigate the influence of RFE and peak force at the end of stretching on the steady-state isometric force following shortening preceded by different stretch speeds. We found that the faster the stretch speed, the greater the peak force at the end of stretching. Greater forces at the end of stretching were also associated with greater amounts of work performed during shortening. The different values for the peak forces at the end of stretch and the work during shortening did not influence the steady-state isometric forces, i.e. force depression (FD), at the end of the stretch-shortening cycles. Therefore, we conclude that the shortening-induced force depression following a SSC depends exclusively on the amount of force enhancement established in the initial stretch phase in situations where the timing and contractile conditions of the shortening phase are kept constant.

It is well accepted that RFE and passive RFE are long-lasting^27,28^. Additionally, it is also well accepted that RFE can be instantly abolished if a muscle is deactivated^1,29^ and passive RFE can be instantly abolished with a quick shortening of sufficient magnitude^27,30^, suggesting that the mechanism behind RFE may have an active and a passive component, respectively^31^. In our current experiments, the steady-state isometric forces following SSCs are greater than for pure shortening contractions (i. e. FD is smaller), but the difference in force between these two conditions is smaller than the pure RFE measured in pure stretch conditions, suggesting that some (but not all) of the RFE is preserved in the SSCs. We speculate that such result could be due to the fact that we lose the passive component of the RFE in SSC (due to the shortening phase). However, the active component may be preserved. Furthermore, it could be that the loss of the passive component of RFE is time-dependent and not instantaneous, and might take 1–2 s in this preparation to fully establish itself.

In previous studies, it had been reported that FD following shortening was not affected by previous stretching of the cat soleus muscle, whereas RFE was influenced in a dose-dependent manner by shortening prior to stretch, suggesting that the mechanisms underlying FD and RFE are different^17,32^. The results of these prior studies could not be explained with the rheological muscle model proposed by Forcinito *et al*.^10^, which accounts for history-dependent muscle properties using the engagement of a passive structural element upon activation. However, Fortuna *et al*.^21^ found that stretching preceding shortening affects FD in a time- and speed-dependent manner, supporting the results found previously by Seiberl *et al*.^20^. More specifically, Fortuna *et al*.^21^ manipulated either the time interval (0, 0.5, and 1 s) between stretch and shortening or the shortening speed (15, 20, 30, and 60°/s) following the stretch phase, and suggested that the effects of the RFE diminished as a function of time and were completely abolished when the time between the end of stretch and the end of shortening reached approximately 2 s.

### Transient force values

The force values before stretching (b-STR), at the end of stretching (e-STR), and at the end of shortening (e-SHO) were assessed to ensure a consistent force output among trials. Force before shortening (b-STR) was similar across all trials, as expected. Also, as expected based on the force-velocity relationship^33,34^, force at the end of stretching (e-STR) was greater for the fastest stretch speed (120°/s), followed by 60°/s, and lowest for the slowest stretch speed (15°/s). In contrast, force at the end of shortening (e-SHO) was the same for all SSC conditions, but was significantly higher compared to the pure FD tests that did not include stretching. A higher value of peak force at the end of stretching (e-STR) for increased stretch speed was expected to produce a higher value of force at the end of shortening (e-SHO), but this result was not found probably due to high subject variability. One might expect that the higher force values at the end of stretching for the higher stretch speeds (e-STR) would result in higher forces at the steady-state following the SSCs, thus resulting in less FD. However, the force at the end of shortening (e-SHO) was similar across all SSC conditions, suggesting that the force at the end of stretch in SSCs does not affect FD and does not affect the steady-state force following SSCs.

### Work values

the work performed during shortening was significantly greater for all SSCs compared to the pure FD tests, because force was greater throughout the shortening phase in SSCs compared to the pure FD tests. For pure shortening contractions, it is generally accepted that FD is directly proportional to the work performed in the shortening phase^35–37^. Interestingly, the greater amount of work in the shortening phase of the SSCs did not translate into increased FD, as might have been expected. Therefore, the relationship between “work performed” and “FD” that exists for pure shortening conditions is incomplete and does not apply for SSC conditions. We assume that the contractile conditions and titin-based forces are distinctly different between the pure FD trials and the SSC conditions, and that these differences affect the resulting steady-state isometric forces in ways that are not fully understood at this time.

Force depression has been thought to be caused by a stress-induced inhibition of cross-bridge attachment in the newly formed overlap zone between thick and thin filaments^38^. Also, FD has been found to be related to the amount of work performed during shortening^35^. In classic FD models, the work is performed primarily by active cross-bridge based sources. However, in our SSC experiments, a proportion of the work performed in the shortening phase may come from elastic recoil, which would differ across speeds. In the present study, the total (active and passive) work performed across all SSC conditions was similar, therefore it is possible that the active cross-bridge based work was greater in the slow compared to the fast SSCs. Since FD has been associated with the amount of (active) work during shortening, one would then expect forces to be lower following SSCs with slow stretch speeds compared to fast stretch speeds. However, since we found no difference between the SSC conditions, it could be that the greater forces at the end of stretch offset the presumably lower active work produced in fast SSCs compared to the slow SSCs. Thus, we might not find differences in the isometric steady-state forces following the different SSC conditions not because this force exclusively depends on the amount of RFE, but because the effect of increased force at the end of stretch is offset by the reduced active work in the fast SSCs compared to the slow ones. To address this issue, future studies could compare the work performed by active and passive components during SSCs.

Taken together, the results reported here suggest that neither the peak force at the end of stretching (e-STR) nor the work performed during shortening influence the FD values following a SSC. If this is the case, the “mechanical work theory” that explains the decrease in the steady-state isometric force following active shortening might not apply to force depression in SSCs. Since the RFE at the end of stretching for the different stretch speeds was the same, it appears that FD in SSC might depend exclusively on the amount of RFE established prior to the shortening phase. This proposal needs to be checked by producing conditions in SSCs where the force at the end of stretch is the same, but the RFE is not. If our proposal is correct, then the FD at the end of the SSCs should vary in a predictable way with the RFE after the active stretch phase: a greater RFE should be associated with greater force depression. Such conditions could be achieved by varying the magnitude of stretch (thereby producing different amounts of RFE) while varying the speeds of stretch (thereby producing different forces at the end of the stretch phase). By combining increasing stretch magnitudes (more force at the end of stretch) with decreasing stretch speeds (less force at the end of stretch), it should be possibly to match forces at the end of stretch while the RFE (which only depends on the stretch magnitude but not the stretch speed) differs between the different conditions.
